# Recent Progress in Bi-Based Anodes for Magnesium Ion Batteries

**DOI:** 10.3390/molecules27227751

**Published:** 2022-11-10

**Authors:** Meijia Song, Hui Gao, Zhonghua Zhang

**Affiliations:** 1 School of Energy and Power, Jiangsu University of Science and Technology, Zhenjiang 212000, China; 2 Key Laboratory for Liquid-Solid Structural Evolution and Processing of Materials (Ministry of Education), School of Materials Science and Engineering, Shandong University, Jingshi Road 17923, Jinan 250061, China

**Keywords:** magnesium ion batteries, alloy-type anodes, Bi-based materials, progress and challenges

## Abstract

Rechargeable magnesium ion batteries (MIBs) have attracted increasing interest due to abundant reserves, high theoretical specific capacities and safety. However, the incompatibility between Mg metal and conventional electrolytes, among the most serious challenges, restrains their development. Replacing Mg metal with alloy-type anodes offers an effective strategy to circumvent the surface passivation issue of Mg metal in conventional electrolytes. Among them, Bi has the most potential in Mg storage owing to its unique characteristics. Herein, the advantages/challenges and progress of Bi-based anodes in MIBs are summarized. The theoretical evaluations, battery configurations, electrode designs, electrochemical properties as well as Mg storage mechanisms are summarized and discussed. Moreover, the key issues and some views on the future development of Bi-based anodes in MIBs are provided.

## 1. Introduction

Lithium ion batteries (LIBs) technology is currently at the forefront of electrochemical energy storage with its widespread application in electrical vehicles, portable devices, etc. However, the increasingly depleted Li resources and uneven worldwide distribution will lead to the inevitable cost rise and questionable sustainability. Moreover, Li dendrite growth easily appears during repeated electroplating, especially in the case of uneven electrode surface or excessive current density, which can pose several safety issues [1,2,3]. The need to achieve a safe and cost-effective high-energy system has prompted researchers to explore alternates beyond LIBs. Specially, magnesium ion batteries (MIBs) have triggered extensive attention owing to their attractive characteristics, such as their high theoretical specific capacity (3833 mAh cm^−3^, 2205 mAh g^−1^), abundant raw materials (the atomic abundance of Mg is ≈10^4^-foldhigher than that of Li in the earth crust) and because they are environmentally friendly. More importantly, unlike alkali metal anodes (Li, Na and K) that often suffer from severe dendrite formation, Mg anodes tend to generate a smooth and homogeneous deposition layer during the electroplating process, avoiding dendrite nucleation and growth, and thus resulting in higher security [4,5]. Note that the electrodeposition of Mg anodes is not completely dendrite free under all conditions, which depends heavily on the electrolyte used and current density [6,7]. In general, these appealing characteristics make MIBs quite promising among the next-generation energy storage systems.

Since Aurbach et al., first reported the prototype system of rechargeable magnesium batteries in 2000 [8], some key breakthroughs were accomplished for the research and progress of MIBs. Nevertheless, there are still many obstacles to the construction of practical MIBs. Among the toughest challenges is the severe incompatibility of Mg metal anodes with conventional electrolytes [9,10]. In most conventional electrolytes based on simple Mg salts (Mg(ClO_4_)_2_, Mg(BF_4_)_2_, Mg(HMDS)_2_, Mg(AsF_6_)_2_, etc.) and the usual organic solvents (carbonates, nitriles, lactones, esters, etc.), an insulating passivation layer would form on the surface of Mg metal, directly blocking the subsequent electrochemical reaction [4,11,12,13,14]. Various types of specially designed electrolytes that enable reversible Mg deposition/stripping were investigated and developed, mainly including Grignard reagent-based electrolytes, all-phenyl complex (APC), magnesium aluminum chloride complex (MACC), butyl ethyl complex (BEC), and various boron-centered anions magnesium (BCM)-based, phenolate-based and alkoxide-based electrolytes. The development of these electrolytes sheds light on the progress of MIBs, but poses some problems. The presence of chloride ions in some electrolytes (such as Grignard reagent, APC and MACC) leads to the high corrosion of battery components. Moreover, the high cost, low anodic stability and the difficulty in reaching high coulombic efficiencies of these electrolytes limit their practical application [15,16,17]. From another point of view, the exploration of alternative Mg anode materials is a strategic method to circumvent the passivation film issue on the surface of Mg metal in conventional electrolytes. In recent years, alloy-type anodes have demonstrated enormous potential due to their high theoretical specific capacities, low reaction potentials and compatibility with most conventional electrolytes. Group IIIA, IVA, and VA elements and their derivative alloys (such as Bi-, Sn-, Sb-, In-, Pb- and Ga-based materials) were widely investigated as alloy-type anodes for MIBs and have achieved definite progress in recent years [4,18,19,20]. Among them, Bi is the most attractive element, but many breakthroughs are urgently needed for the further improvement and practical application of Bi-based alloy-type anodes for MIBs.

In this minor review, we mainly focus on the intrinsic advantages/challenges and development/progress of Bi-based alloy-type anodes for MIBs. The influence of structural and compositional design on Mg storage performance of Bi-based anodes is elaborated. Additionally, the reaction mechanisms of different Bi-based system are discussed and summarized in detail. Finally, some outlooks and perspectives are proposed. We hope this review can provide insights and valuable references for the further exploration of Bi-based materials in MIBs.

## 2. Advantages and Challenges of Bi-Based Alloy-Type Anodes

Based on the alloying reaction of Bi-Mg (2Bi + 3Mg^2+^ + 6e^−^ ↔ Mg_3_Bi_2_), Bi anodes can deliver a theoretical specific capacity of 385 mAh g^−1^ at 0.23 V vs. Mg^2+^/Mg in MIBs, which exceeds that of the commercial graphite in LIBs (372 mAh g^−1^). More encouragingly, the theoretical volumetric specific capacity of Bi-Mg alloy (3783 mAh cm^−3^) is comparable to that of the Mg metal (3833 mAh cm^−3^), which is of vital importance to portable devices [21]. Noticeably, the overall energy stored may be compromised due to the higher reaction potential of Bi electrodes compared to Mg metal. Furthermore, a most attractive feature of Bi anodes is the rapid mobility of Mg^2+^. Generally, the kinetics of divalent Mg^2+^ in many anodes is usually much more sluggish than those of monovalent ions (Li^+^, Na^+^). Notably, theoretical studies have shown that the calculated Mg^2+^ diffusivities in Mg_3_Bi_2_ (3.9 × 10^−10^ cm^2^ s^−1^)/Bi (5.9 × 10^−14^ cm^2^ s^−1^) are even several orders of magnitude larger than/comparable to the calculated Li^+^ ion diffusivity in Bi (3.9 × 10^−14^ cm^2^ s^−1^) [22], indicating that Bi/Mg_3_Bi_2_ may be able to serve as a favorable transport medium of Mg^2+^, promoting the electrochemical reactivity of other less active elements. However, the insertion of Mg into Bi (a rhombohedral crystal structure) to form Mg_3_Bi_2_ (a hexagonal crystal structure) will lead to substantial internal stress, causing the serious volume variations and capacity fading of electrodes during the repeated discharge/charge processes. Such issues need to be urgently addressed for the development and progress of Bi-based alloy-type anodes in MIBs.

## 3. Progress of Bi Anodes

In this part, we mainly take the development time course of Bi-based electrodes as the main line, and discuss the electrode structural modifications, composite materials, theoretical simulations and reaction mechanisms of Bi-based alloy-type anodes.

**Figure 1:**
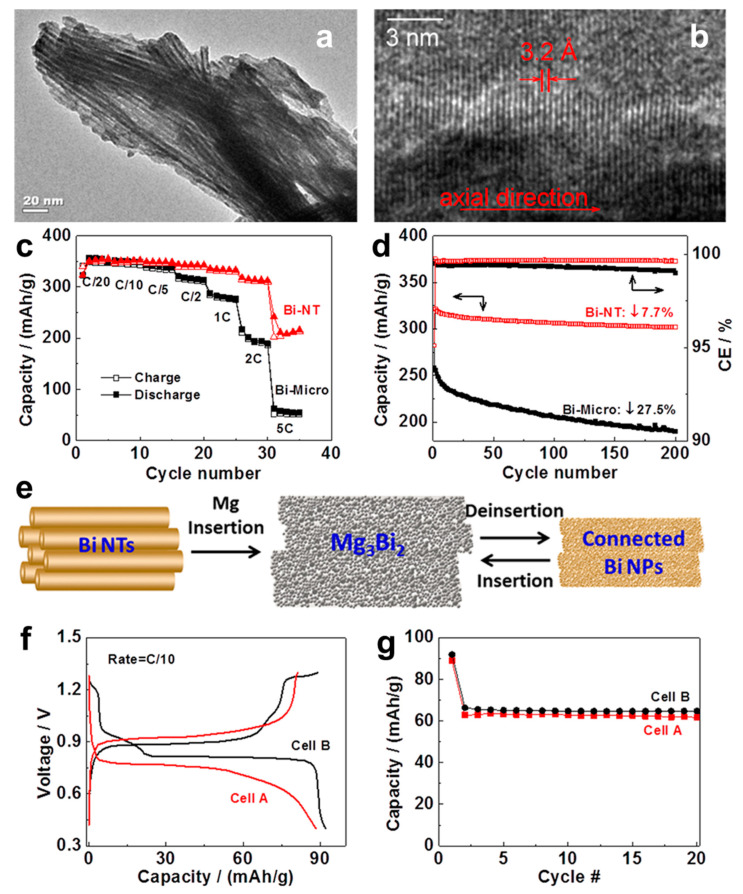
(**a**) TEM and (**b**) HRTEM images of Bi-NTs. (**c**) Rate performance (the hollow triangle/implement triangles represent charge/discharge of Bi-NT) and (**d**) cycling stability of Bi-NTs and micro-sized Bi as anodes for MIBs (the two arrows in different directions represent capacity and Coulombic efficiency respectively). (**e**) Schematic illustration of the structural transformation of Bi-NT electrode during the discharge/charge processes. (**f**) Discharge/charge profile and (**g**) cycling stability of an Mg_3_Bi_2_-Mo_6_S_8_ cell. Cell configuration: (A) Mg_3_Bi_2_/0.4 M Mg(TFSI)_2_-diglyme/Mo_6_S_8_, (B) Mg_3_Bi_2_/0.1 M Mg(BH_4_)_2_-1.5 M LiBH_4_-diglyme/Mo_6_S_8_. Reproduced with permission [24]. Copyright 2014, American Chemical Society.

Arthur et al. [23] have first reported the electrodeposited Bi film anodes for MIBs in coin cells versus Mg metal using ethylmagnesium chloride (EtMgCl)-diethylaluminum chloride (Et_2_AlCl)/tetrahydrofuran (THF) electrolyte. The electrodeposited Bi film anodes showed good cycling performance, maintaining a specific capacity of 222 mAh g^−1^ after 100 cycles at 1C from a maximum specific capacity of 247 mAh g^−1^ at the 20th cycle. (C is the theoretical specific capacity of electrodes (e.g., 385 mAh g^−1^). 1C (e.g., 385 mA g^−1^) is the current density at which the battery will be fully discharged or charged in 1 h of operation; 2C (e.g., 770 mA g^−1^) is the rate at which the battery is fully discharged or charged in 30 min). Ex situ X-ray diffraction (XRD) results demonstrated that the Bi film anodes followed a reversible magnesium storage process of 2Bi + 3Mg^2+^ + 6e^−^ ↔ Mg_3_Bi_2_. Moreover, the electrodeposited Bi film anodes exhibited good compatibility with a conventional electrolyte (Mg(N(SO_2_CF_3_)_2_)_2_/acetonitrile (AN)), which is an essential first step towards extending the voltage window for MIBs. Afterwards, the high-performance Bi nanotubes (Bi-NTs) as MIB anodes were developed by Shao et al. [24]. Bi-NTs were synthesized through a hydrothermal reaction method by reacting a mixture of BiCl_3_ and zinc (Zn) powders with diluted hydrochloric acid. The pristine Bi-NTs present a bundle structure with uniform diameters of approximately 8 nm and wall thickness of approximately 2 nm (Figure 1a,b), stemming from the van der Waals interaction between small tubes. The Bi-NT electrodes were tested in the half cells versus Mg metal utilizing the 0.1 M Mg(BH_4_)_2_-1.5 M LiBH_4_/diglyme electrolyte, accompanied with the control electrodes using Bi microparticles (Bi-Micro, approximately 100 μm) for comparison. The Bi-NT electrodes delivered an excellent rate performance with the capacity maintained at 216 mAh g^−1^ at 5C, while the capacity of Bi-micro-electrodes dramatically decreased, with the current densities increasing, retaining only 51 mAh g^−1^ at 5C (Figure 1c). Moreover, the cycling stability of Bi-NTs (303 mAh g^−1^ after 200 cycles) was also substantially better than that of Bi-Micro (only 188 mAh g^−1^ after 200 cycles), as shown in Figure 1d. The significantly improved rate and cycling performance of Bi-NT anodes demonstrate that the tubular structure is very effective for promoting Mg^2+^ transport and mitigating the volume expansion/shrinkage of Bi during the discharge/charge processes. The simple biphasic transition between Bi and Mg_3_Bi_2_ was verified by ex situ XRD. For Bi-NT anodes, the nanotubes were converted into interconnected nanoparticles after Mg insertion and roughly retained the overall nanotube morphology (Figure 1e). Noticeably, the existence of hollow space in nanotubes may be able to guarantee the highly connected Bi nanoparticle structure along the Bi nanotubes and high conductivity between these nanoparticles during repeated discharge/charge cycles, endowing the Bi-NT anodes with outstanding structural integrity and Mg storage performance. However, the Bi-Micro anodes, without hollow space inside, just pulverized and lost the electronic connection during the discharge/charge processes, leading to the poor electrochemical performance. On the other hand, the full cell coupled with the premagnesiated Bi-NT anode (Mg_3_Bi_2_) and Mo_6_S_8_ cathode in 0.4 M Mg(TFSI)_2_/diglyme conventional electrolyte, exhibited stable cycling performance and similar discharge/charge behavior to that in 0.1 M Mg(BH_4_)_2_-1.5 M LiBH_4_/diglyme electrolyte (Figure 1f,g), demonstrating the well compatibility of Bi anodes with conventional electrolytes. This work achieves a profound improvement for Bi anodes in MIBs and opens up a new approach to develop electrode materials.

**Figure 2:**
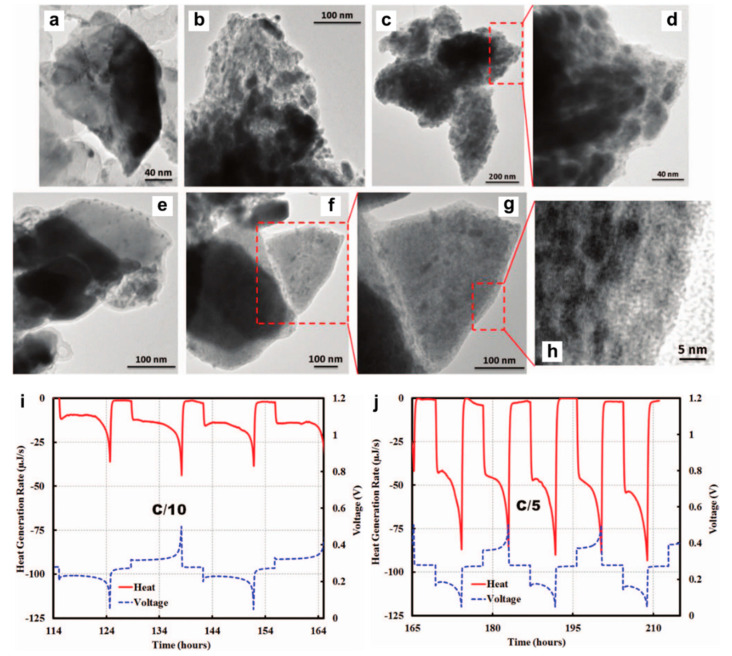
TEM images depicting the structure transformations of Bi during the processes of magnesiation/demagnesiation, (**a**) pristine Bi, (**b**) 50% magnesiated Bi, (**c**,**d**) 100% magnesiated Bi (i.e., Mg_3_Bi_2_), (**e**) 50% demagnesiated Mg_3_Bi_2_, and (**f**–**h**) 100% demagnesiated Mg_3_Bi_2_ (i.e., Bi). In situ heat generated by the Mg/Bi coin cell during magnesiation/demagnesiation using IMC at (**i**) C/10 and (**j**) C/5 rate, respectively. Reproduced with permission [25]. Copyright 2015, Institute of Physics.

**Figure 3:**
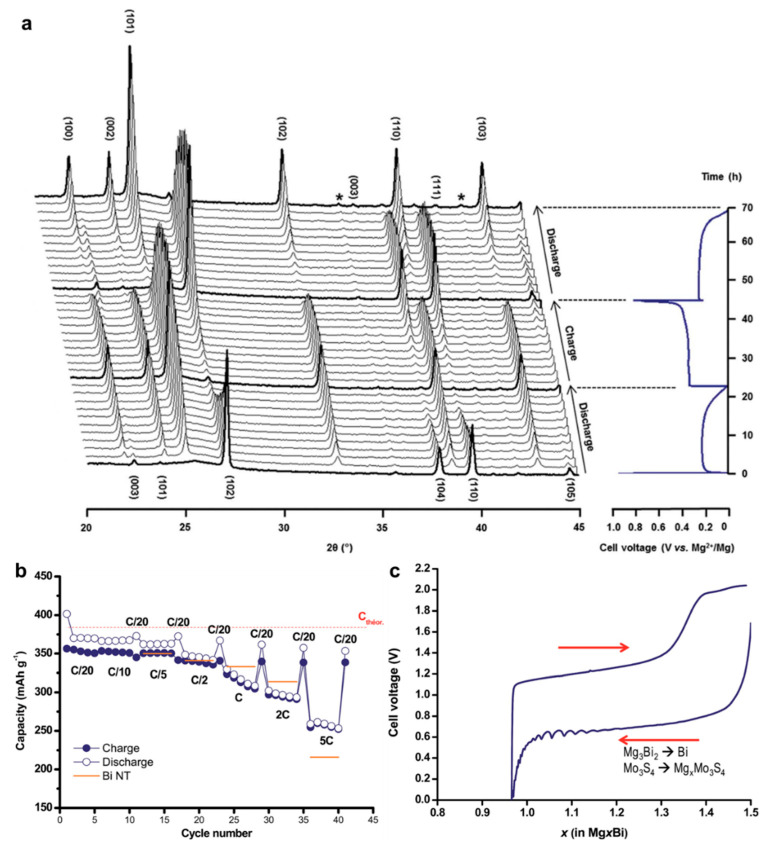
(**a**) Operando XRD characterization of the first discharge, charge and second discharge of a Bi/Mg battery. The peak (*) corresponds to the diffraction peak of poly−tetrafluoroethylene (PTFE). (**b**) Rate performance of micrometric Bi powder electrode with current densities from C/20 to 5C. The performance is also compared with results obtained from Bi-NT. (**c**) Electrochemical behavior of the full cell consisting of the Mo_3_S_4_ cathode and the as-prepared ball-milled Mg_3_Bi_2_ anode in the 0.5 M Mg(TFSI)_2_/diglyme electrolyte. Reproduced with permission [26]. Copyright 2015, The Royal Society of Chemistry.

Both thin films and nanotube structures are advantageous for Bi anode to exert its Mg storage capability. As for the intrinsic nature of Bi element towards Mg storage, Benmayza et al. [25] have investigated the morphological changes and thermal stability of the commercial Bi anodes during the magnesiation/demagnesiation processes. The electrolyte was the 0.25 M EtMgCl-(Et_2_AlCl)_2_/THF and the polished Mg metal foil was used as the counter/reference electrodes. The commercial Bi anodes could deliver reversible capacities over 300 mAh g^−1^ and high coulombic efficiencies of >98%. Transmission electron microscopy (TEM) characterizations indicate the pulverization of the pristine Bi material into small particles during the magnesiation processes (Figure 2a–d) and the formation of an obvious amorphous layer around the “reforming” Bi materials after charge (Figure 2e–g). However, the functionality of the amorphous layer towards the Mg storage of Bi anodes was not explained. Moreover, after complete demagnesiation, the nanocomposite morphology composed of the congealed state (5–10 nm particles with high crystallinity) and amorphous region (lighter contrast in the TEM image) was observed (Figure 2h), which may have a positive effect on the performance of Bi anodes at higher C-rates. In addition, isotherm micro-calorimetry (IMC) results of this system demonstrated the low-heat generation in Bi during the discharge/charge processes (Figure 2i,j). This property in combination with the absence of a noted SEI may be the essential conditions for the chemical and thermal stabilities of Bi anodes in MIBs. Afterwards, Murgia et al. [26] have revisited the electrochemical behaviors of commercial Bi powders by ball milling in a half-cell with Mg metal as the counter/reference electrodes and 0.35 M EtMgCl-Et_2_AlCl/THF as the electrolyte. Operando XRD results underlined the simple biphase reaction mechanism from Bi to Mg_3_Bi_2_ without any intermediate phases or amorphization process (Figure 3a). Moreover, the rate capability of the micrometric Bi powder electrodes was in line with that obtained with Bi-NT electrodes (Figure 3b). Noticeably, the micrometric Bi powder electrodes delivered high capacities at C/20, which could be remained constant for each C/20 cycling following a few cycles at high rates. This reason was explained by the fact that the electrochemical grinding could progressively pulverize the pristine micrometric powder and act as an in situ self-nanostructuration, which accounted for the excellent Mg storage performance of Bi anodes in MIBs. On the other hand, the mechanochemically prepared Mg_3_Bi_2_ showed good compatibility with the 0.5 Mg(TFSI)_2_/diglyme electrolyte in a prototype Mg ion full cell (Figure 3c). These investigations further confirm the interest in the development of Bi-based anodes in MIBs.

**Figure 4:**
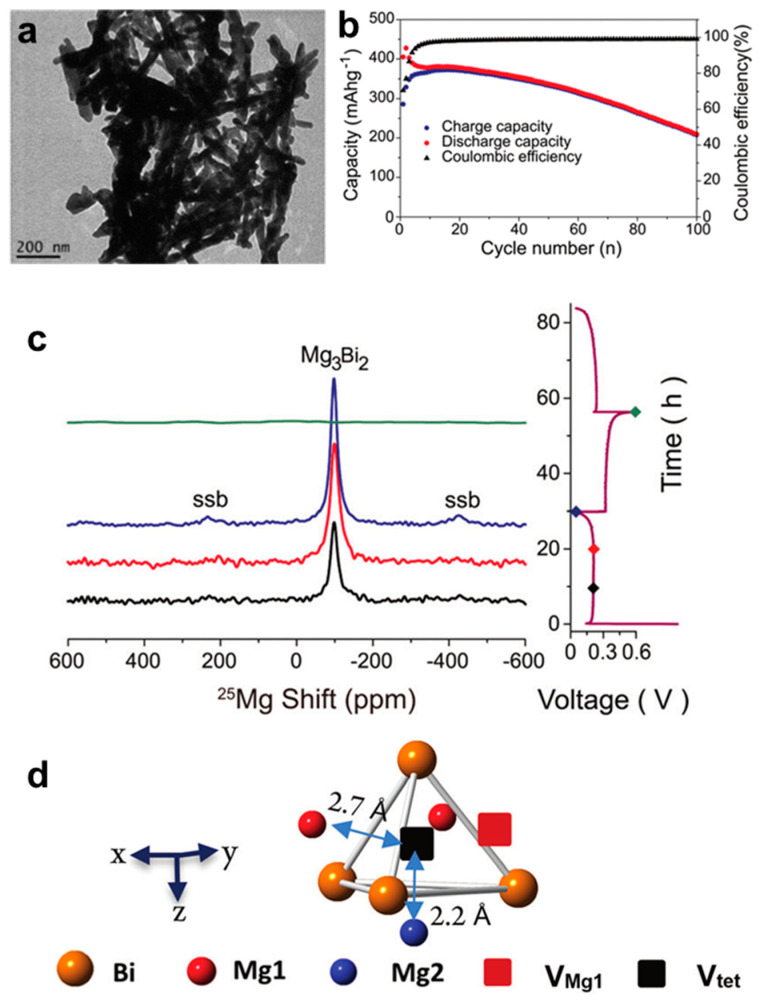
(**a**) TEM image of the Bi nanowires. (**b**) Cycling stability of the Bi nanowire anodes at C/2 rate. (**c**) Quantitative ^25^Mg NMR spectra of Bi anode at various states of charge (with color corresponding to the points shown on the electrochemistry profile in the right) at a spinning rate of 14 kHz. Spinning sidebands labeled in the spectra as ssb. (**d**) Schematic structure of Mg_3_Bi_2_ with V_oct_ (red square) and V_tet_ (black square). Reproduced with permission [27]. Copyright 2017, The Royal Society of Chemistry.

To further enhance the electrochemical performance of Bi anodes in MIBs and deeply understand the Mg storage mechanism, Liu et al. [27] have prepared the Bi nanowire electrodes for MIBs via the reduction of aqueous BiCl_3_ with Zn. TEM result shows that the samples are composed of individual nanowires with diameters of approximately 40 nm and lengths of approximately 300 nm (Figure 4a). This Bi/Mg cells delivered high cycling stability (207 mAh g^−1^ after 100 cycles) and stable coulombic efficiency with an organohaloaluminate electrolyte (Figure 4b). Furthermore, the two-phase reaction between Bi and Mg_3_Bi_2_ during the discharge/charge processes was confirmed by the ^25^Mg nuclear magnetic resonance (NMR) spectra (Figure 4c). Further, the ^25^Mg variable temperature (VT) NMR experiments revealed the fast exchange between the two Mg sites inside the Mg_3_Bi_2_ structure and a hop mechanism involving Mg1 and Mg2 exchanging via an interstitial tetrahedral site (Figure 4d). This work has important implications for understanding the Mg storage mechanism of Bi anodes and exploring the diffusion pathway of Mg^2+^. Afterwards, Kravchyk et al. [28] have demonstrated that the colloidal Bi nanocrystals can be used as the promising anode materials for MIBs, which delivered a stable capacity of 325 mAh g^−1^ over 150 cycles at 770 mA g^−1^ in the Mg(BH_4_)_2_-LiBH_4_/diglyme electrolyte. Density functional theory (DFT) simulations combined with ex situ XRD characterizations revealed the simultaneous formation of the low-temperature trigonal structure (α-Mg_3_Bi_2_) and the high-temperature cubic structure (β-Mg_3_Bi_2_) during the magnesiation of Bi nanocrystal electrode, confirming the high stability of this reversible alloying reaction. Narumoto et al. [29] have employed a simple electrodeposition method to prepare Bi thin films as anodes for MIBs and regulated the surface structure and film thickness by changing experimental parameters (deposition current densities from 10 to 50 mA cm^−2^). It was found that the cycling performance of the Bi electrodes deteriorated with the increase in film thickness. Further, the Bi anode with the thickness of 1.5 μm, which was deposited at a current density of 10 mA cm^−2^, could deliver good cycling stability with the capacity maintained at 100.8 mAh g^−1^ after 50 cycles. According to the above results, the nano-size of electrode materials and the structural regulation of different dimensions such as films, tubes and wires, are vital to improving the Mg storage performance of Bi-based anodes. 

In addition to the structural modification of electrodes, constructing composite materials is also an effective strategy to improve the electrochemical performance of Bi anodes based on the favorable electronic conductivity and buffer matrix. DiLeo et al. [30] first fabricated the novel Bi-carbon nanotube (Bi-CNT) composite electrodes via electrodeposition of nano-sized Bi on CNT substrates by a cyclic voltammetric method. The Bi-CNT composite electrodes exhibited the quasi-reversible Mg electrochemistry in acetonitrile-based electrolyte containing 0.5 M magnesium perchlorate (Mg(ClO_4_)_2_) and 0.5 M dipropylene glycol dimethyl ether (DPGDME), with initial capacities exceeding 180 mAh g^−1^. These results confirmed the good compatibility of Bi anodes with conventional electrolytes and may hold promise for the further development of MIBs in non-corrosive electrolytes. Noticeably, the capacities of the Bi-CNT electrode attenuated rapidly during repeated cycling, indicating this MIB system needed further optimization and improvement. 

**Figure 5:**
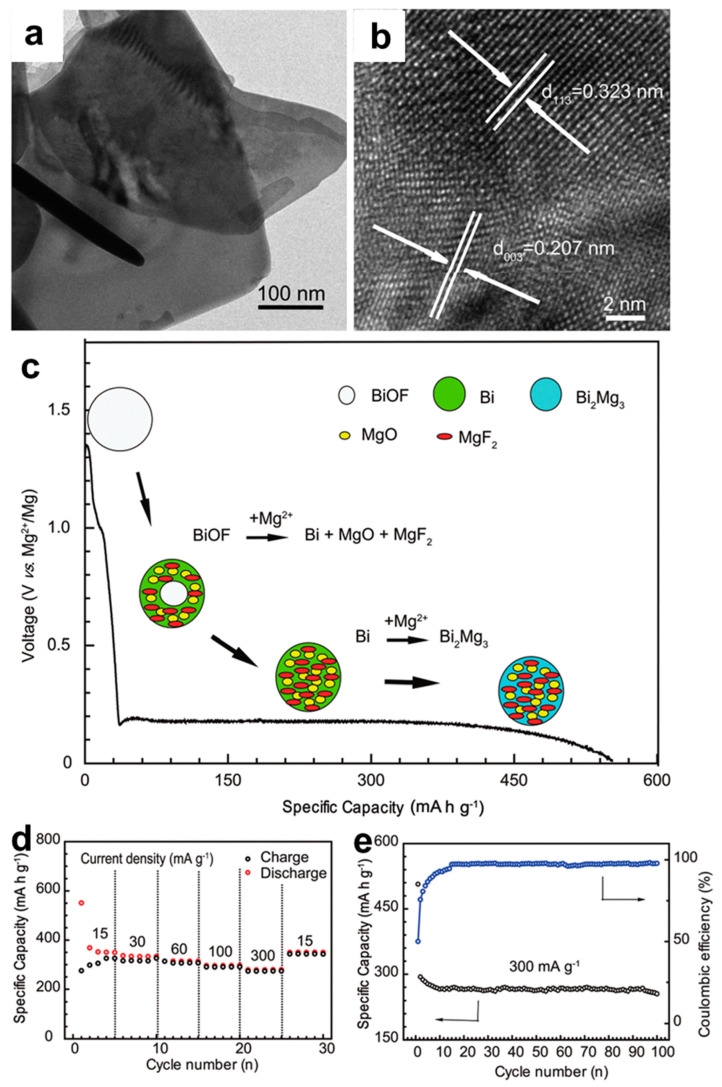
(**a**) TEM and (**b**) HRTEM images of the BiOF nanosheets. (**c**) The schematic illustration of the phase evolution reaction during the discharge process. (**d**) The rate performance and (**e**) cycling stability of the cells using the BiOF electrodes. Reproduced with permission [31]. Copyright 2018, The Royal Society of Chemistry.

Aiming to further alleviate the capacity decay through the limitation of volume variation in Bi-based composite materials, Wang et al. [31] have fabricated bismuth oxyfluoride (BiOF) nanosheets via a simple solvothermal method and investigated their Mg storage properties with the 0.25 M Mg(AlCl_2_BuEt_2_)_2_/THF electrolyte. TEM and high-resolution TEM (HRTEM) images showed that the samples are comprised of the square-like sheets, and two d spacing values of ~0.207 and 0.323 nm can be indexed to the (003) face of BiOF and (113) face of Bi_2_O_3_, respectively, revealing a heterojunction structure of BiOF and Bi_2_O_3_ (Figure 5a,b). Ex situ XRD analyses coupled with X-ray photoelectron spectroscopy (XPS) results elaborated that a conversion reaction from BiOF to metallic Bi occurred during the first magnesiation process, followed by the reversible alloying reaction from Bi to Mg_3_Bi_2_ in the subsequent cycles (Figure 5c). Benefiting from the space confinement of in situ conversion reaction, the BiOF electrodes exhibited excellent electrochemical performance (Figure 5d,e), especially the superior cycling stability (capacity retention >96% at 300 mA g^−1^ after 100 cycles).

**Figure 6:**
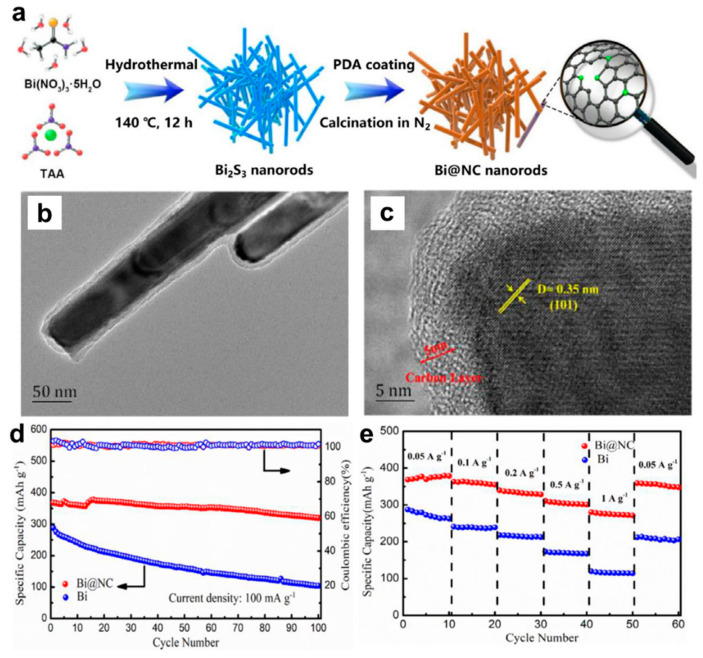
(**a**) Schematic illustration of the formation process of Bi@NC. (**b**) TEM and (**c**) HRTEM images of the Bi@NC. (**d**) Cycling stability and (**e**) rate performance of the Bi and Bi@NC electrodes. Reproduced with permission [33]. Copyright 2021, Elsevier B.V.

**Figure 7:**
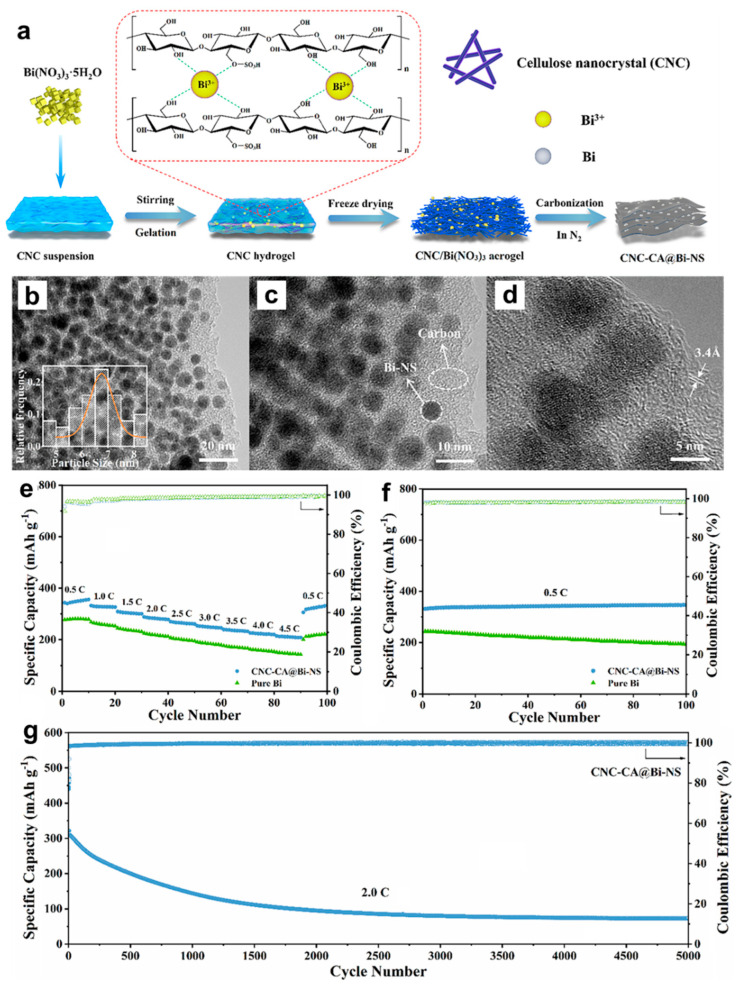
(**a**) Schematic illustration of the fabrication process of CNC-CA@Bi-NS. (**b**–**d**) HRTEM images of CNC-CA@Bi-NS at different magnifications. (**e**) Rate performance and (**f**) cycling stability of pure Bi powder and CNC-CA@Bi-NS electrodes. (**g**) Long-term cycling stability of the CNC-CA@Bi-NS electrode at 2.0 C for 5000 cycles. Reproduced with permission [34]. Copyright 2023, Elsevier B.V.

Afterwards, researchers have also developed other effective Bi-based composite anodes to improve their Mg storage performance. Penki et al. [32] have prepared the Bi nanoparticle-anchored reduced graphene oxide (Bi/RGO) by in situ reduction of GO and Bi^+^ under solvothermal condition at 100 °C under a N_2_ atmosphere, which was used as anodes in MIBs and expected to improve the electrochemical performance by increasing the electronic conductivity and reducing the mechanical stress upon cycling. The nanocomposite of 60% Bi:40% RGO delivered much better cycling and rate performance than pure Bi, indicating that Bi/RGO nanocomposite appeared to be a promising high-capacity anode for MIBs. Cen et al. [33] have fabricated a novel bismuth-carbon composite with bismuth nanorods anchored in nitrogen-doped mesoporous carbon matrix (Bi@NC) as anodes for MIBs, through carbonizing the dopamine-coated bismuth metal precursors (Figure 6a). The TEM results show that the amorphous carbon shell with the thickness of approximately 5 nm was uniformly coated on the Bi nanorod surface and the average diameter of the Bi@NC nanorods was nearly 50 nm (Figure 6b,c). Compared with the pure Bi electrodes, the Bi@NC nanorod electrodes exhibited high specific capacity (360 mAh g^−1^ at 100 mA g^−1^), good cycling stability (87% capacity retention after 100 cycles) and excellent rate performance (275 mAh g^−1^ at 1 A g^−1^), as presented in Figure 6d,e. The improved Mg storage performance of Bi@NC nanorod electrodes could be attributed to the synergetic effect of the unique architecture and N-doped carbon, which effectively shortened the transportation path of Mg^2+^, increased the electronic conductive/surface area and alleviated the mechanical strain of Bi anodes during the discharge/charge processes. Recently, Cheng et al. [34] have prepared a unique Bi nanospheres homogenously anchored in cellulose nanocrystal-derived carbon aerogel (CNC-CA@Bi-NS) hybrid as anodes for MIBs through ion-induced gelation and in situ thermal reduction processes (Figure 7a). The HRTEM results of CNC-CA@Bi-NS demonstrated that a large number of regular Bi nanospheres with the diameter of 4~9 nm were uniformly anchored into the 3D architecture porous carbon framework without aggregation (Figure 7b–d). Benefiting from this favorable structure, the CNC-CA@Bi-NS electrodes exhibited much better rate performance (214 mAh g^−1^ at 4.5 C) and cycling stability (346 mAh g^−1^ at 0.5 C (90% of theoretical capacity) after 100 cycles) than the pure Bi powder electrodes (Figure 7e,f). More importantly, the CNC-CA@Bi-NS electrodes delivered an unprecedented long-term cycling capability with a high coulombic efficiency near to 100% at 2C after 5000 cycles, as shown in Figure 7g. However, the remaining reversible capacity is only 73.0 mAh g^−1^ after 5000 cycles with the capacity retention of approximately 22.5%, indicating that the capacity decay during the initial 1000 cycles needs to be further addressed. This work provides important inspiration for exploring the novel and advanced Bi-based electrodes for MIBs.

Combined with the theoretical calculation, the Mg storage properties of Bi-based anodes can be understood more thoroughly. Jin et al. [35] have illustrated the potential of Bi anodes in MIBs based on DFT calculation. The diffusion barrier for an isolated Mg^2+^ in Bi is 0.67 eV, and there is no apparent variation of the diffusion barrier in Bi lattice when an extra Mg^2+^ emerges near the diffusing Mg^2+^, showing that Bi can be an excellent candidate as alloy-type anodes for MIBs with fast discharge/charge rates. Based on the first-principles molecular dynamics study, Jung et al. [22] have proposed that the thermodynamic stability of crystalline Mg_3_Bi_2_ (c-Mg_3_Bi_2_) is much higher than that of amorphous Mg_3_Bi_2_ (a-Mg_x_Bi), resulting in the complete c-Bi/c-Mg_3_Bi_2_ two-phase reaction without the formation of amorphous phases usually observed during alloying reaction. Such results provide an atomic-level strategy for understanding the Mg storage mechanism and Mg^2+^ transports in Bi anodes. Moreover, Jin et al. [36] have studied the surface stability and surface adsorption/intercalation of Bi anodes in MIBs using first-principle calculation. The surface energy is 0.31 J m^−2^, demonstrating the Bi (111) surface is stable. Further, the diffusion energy barriers are 0.37–0.54 eV for the diffusion of Mg inside Bi, accompanied with a large barrier of 1.27 eV for the diffusion of Mg from the surface to the subsurface of Bi, which indicates that the surface modification is needed to further enhance the Mg storage performance of Bi anodes. On the other hand, Hattori et al. [37] have studied the mechanism of Mg^2+^ alloying reaction into Bi utilizing the Mg(TSFI)_2_/acetonitrile (AN) and Mg(TFSI)_2_/2-methyltetrahydrofuran (2-MeTHF) electrolytes, through combining the experiments and DFT calculations. The results demonstrate that Bi anodes have good compatibility with the Mg(TFSI)_2_/AN electrolyte. However, the reversible alloying reaction cannot occur in Mg(TFSI)_2_/2-MeTHF, indicating that reducing the interaction between Mg^2+^ and anions is important for ensuring the compatibility of Bi anodes in conventional electrolytes.

These interesting findings and understandings motivate the extensive explorations on Mg_3_Bi_2_ alloy anodes in MIBs. Tan et al. [21] have reported a facile direct alloying strategy to fabricate nanoclustered Mg_3_Bi_2_ alloy-type anodes to construct the high-performance half/full cells. The synthesized MB-650 (the alloying temperature of Mg_3_Bi_2_ at 650 °C) anodes delivered a high reversible specific capacity (360 mAh g^−1^) with stable cycling performance (90.7% capacity retention over 200 cycles) and high coulombic efficiency (average 98%) in the LiCl-APC electrolyte. Significantly, the full cells coupled with the MB-650 anode and high-voltage Prussian Blue cathode, exhibited superior cycling stability (88% capacity retention over 200 cycles at 0.2 A g^−1^) and excellent rate capability (103/58 mAh g^−1^ at 0.1/2.0 A g^−1^) using the non-corrosive electrolyte, demonstrating the great potential of Mg_3_Bi_2_ electrodes for the application of MIBs in grid-scale energy storage. Matsui et al. [38] have also demonstrated that the Mg_3_Bi_2_ showed excellent electrochemical activity in three type electrolytes containing Mg(TFSA)_2_/AN, Mg(TFSA)_2_/butylmethyltriglyme (BuMeG_3_) and Mg(TFSA)_2_/dimethoxyethane (DME), which interprets that no passivation occurs at the surface of Mg_3_Bi_2_ even with the formation of MgF_2_ layer. The stability test of Mg_3_Bi_2_ further suggested that the reversibility of the intermetallic alloy anode in electrolytes is not only dependent on the passivation-free surface, but also determined by the reaction kinetics. These results illustrate that the formation of Mg_3_Bi_2_ alloy is an effective strategy to settle the passivation issue of anode surface in conventional electrolytes. Recently, Sagane et al. [39] have pointed out that the reactivity of the Mg_3_Bi_2_ electrode will be mainly affected by the activity of Mg^2+^, rather than the MgF_2_-based surface film. Thus, the de-solvation process will be the key factor for the Mg^2+^ insertion/extraction into the Mg_3_Bi_2_ electrode. Assadi et al. [40] have investigated the insertion/extraction process of Mg^2+^ in Mg_3_Bi_2_ using the DFT calculations. Based on the calculated results, Mg^2+^ first vacated the octahedral sites, and then diffused through the tetrahedral sites during the magnesiation/demagnesiation processes. Moreover, the spin-orbit coupling could significantly reduce the formation energy of Mg vacancy, but had negligible effect on the diffusion barriers. These results could provide deeper insight into the Mg^2+^ migration process and (de)alloying mechanism of Mg_3_Bi_2_.

In terms of the good electrochemical behavior of Mg_3_Bi_2_ in MIBs, Meng et al. [41] reported a first proof-of-concept rechargeable Mg ion/S battery with Mg_3_Bi_2_ alloy anode. The Mg_3_Bi_2_/S batteries were evaluated in the Mg(TFSI)_2_/DME electrolyte, exhibiting excellent electrochemical performance at elevated current densities with much smaller overpotentials and higher specific capacities compared with the counterpart Mg/S system. The absence of corrosive chloride species and unnecessary preliminary conditioning treatment of the Mg_3_Bi_2_/S system may offer important references for the future practical application. 

**Figure 8:**
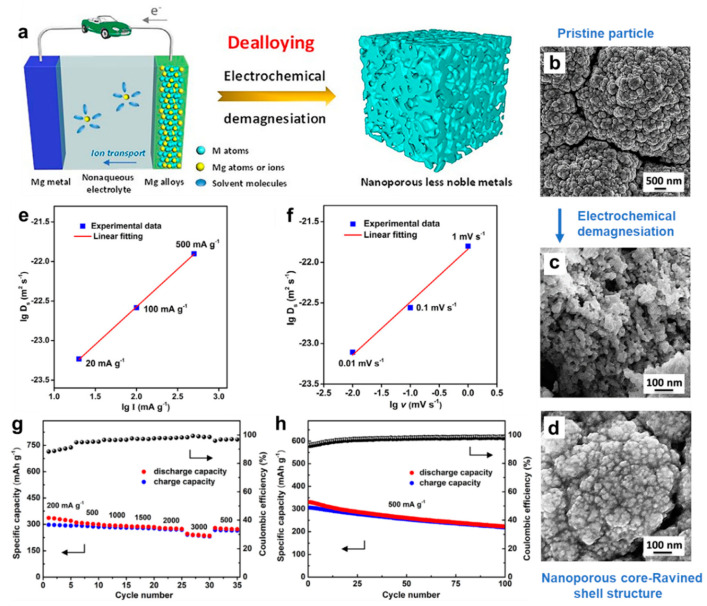
(**a**) Schematic illustration showing the charging-induced dealloying strategy involving electrochemical demagnesiation in MIBs. (**b**–**d**) SEM images of the Mg_3_Bi_2_ film before and after electrochemical demagnesiation. The logarithm of surface diffusivity of Bi adatoms versus (**e**) the logarithm of current density and (**f**) the logarithm of scan rate. (**g**) Rate performance and (**h**) cycling stability of the obtained nanoporous Bi electrodes. Reproduced with permission [42]. Copyright 2021, Elsevier B.V.

From the point of view of electrochemical dealloying of Mg_3_Bi_2_, Niu et al. [42] have investigated the charge process and associated structural evolution of the magnetron-sputtered Mg_3_Bi_2_ thin film in the APC electrolyte through in situ and ex situ characterizations. The basic principle, charging-induced dealloying process and structural evolution of the Mg_3_Bi_2_ film are presented in Figure 8a–d. The experimental results show that the microstructures and length scales of nanoporous Bi can be easily regulated by changing electrochemical parameters. There exists a good linear correlation between the surface diffusivities of Bi and applied current densities/potential scan rates on a logarithm scale (Figure 8e,f). Noticeably, the surface diffusivity for electrochemical dealloying in the APC electrolyte is 4–5 orders of magnitude slower than that for chemical dealloying in the aqueous solution, leading to the refinement of the ligament size of Bi anodes. As anodes in MIBs, the obtained nanoporous Bi delivered satisfactory electrochemical performance (Figure 8g,h) and showed decent compatibility with conventional electrolytes. Therefore, a general charging-induced dealloying strategy in MIBs was proposed to fabricate other nanoporous metals in the nonaqueous electrolyte, which has many unique advantages over the traditional chemical dealloying process.

**Figure 9:**
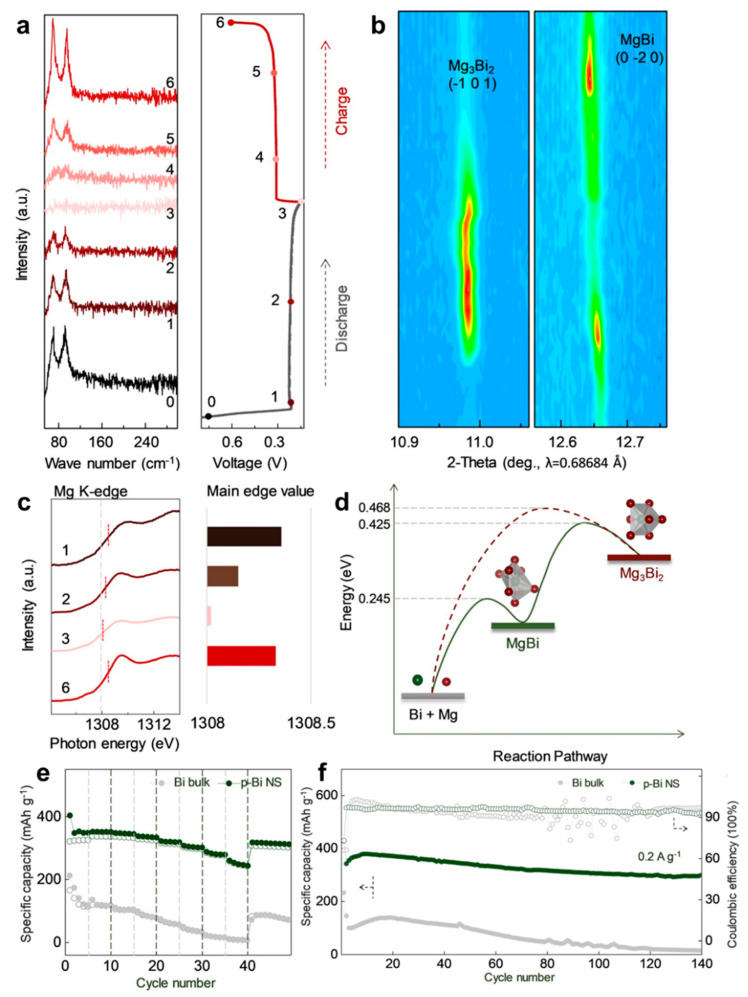
(**a**) The ex situ Raman spectra of p-Bi NS in the initial cycle and the corresponding discharge/charge profile. (**b**) Contour maps of synchrotron-based operando XRD analyses in the initial cycle. (**c**) Normalized Mg K-edge NEXAFS spectra. (**d**) Illustration of computed energy barrier in different reaction pathways with or without an intermediate state. (**e**) Rate performance and (**f**) cycling stability of the Bi bulk and p-Bi NS electrodes. Reproduced with permission [43]. Copyright 2020, Wiley-VCH Verlag GmbH & Co. KGaA.

For clarifying the Mg storage mechanism of Bi anodes, Xu et al. [43] have recently proposed a novel view on the electrochemical reaction processes of Bi anodes, which differs from the previously reported reversible two-phase reaction of Bi ↔ Mg_3_Bi_2_. The mesoporous Bi nanosheets (p-Bi NS) were fabricated by a facile hydrothermal approach and served as a model for the exploration of the reaction mechanism of Mg/Bi systems in MIBs. The systematic spectroscopy investigation was conducted by combining synchrotron-based operando XRD, near-edge X-ray absorption fine structure (NEXAFS) and Raman measurements. In terms of the above-mentioned results, the p-Bi NS electrodes followed a reversible two-step (de)alloying procedures of Bi ↔ MgBi ↔ Mg_3_Bi_2_, where the intermediate phase (MgBi) was first captured during the magnesiation/demagnesiation processes (Figure 9a–c). Moreover, the DFT calculations further confirmed the high electronic conductivity of MgBi and reduced energy barrier under the reaction path of Bi ↔ MgBi ↔ Mg_3_Bi_2_ (Figure 9d), which could accelerate the reaction kinetics and mitigate the significant volume variations during cycling. Benefiting from these properties, the p-Bi NS anodes exhibited much better rate performance (351/247 mAh g^−1^ at 0.1/2.0 A g^−1^) and cycling stability (297 mAh g^−1^ after 140 cycles) than Bi bulk anodes (Figure 9e,f). These findings could deepen the understanding upon the (de)magnesiated mechanism and advance material design principle of Bi anodes in MIBs.

**Figure 10:**
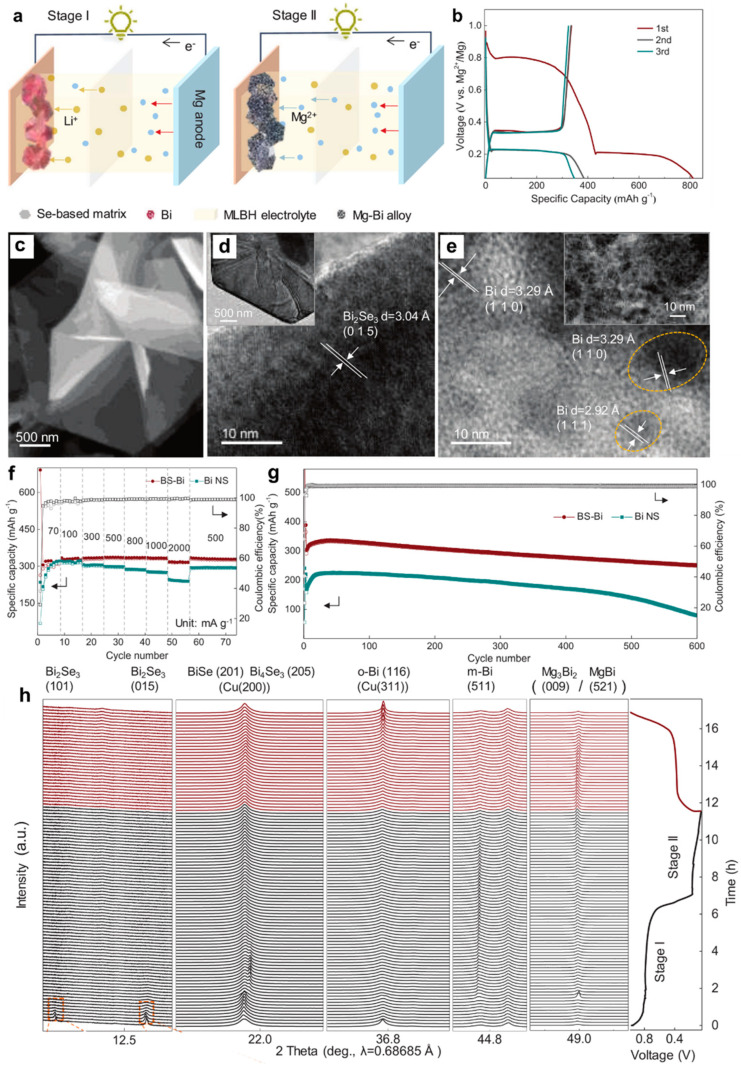
(**a**) Schematic illustration of the two-stage working mechanism of the BS-Bi electrodes. (**b**) Discharge/charge curves of BS-Bi electrode at the current density of 0.1 A g^−1^. (**c**) SEM and (**d**) TEM images of the Bi_2_Se_3_ nanosheets. (**e**) HRTEM image of nanostructured m-Bi obtained from the initial discharge of Bi_2_Se_3_ at a cut-off voltage of 0.75 V. (**f**) Rate performance and (**g**) cycling stability of the BS-Bi and Bi NS electrodes. (**h**) Operando synchrotron XRD patterns of the BS-Bi electrode and the corresponding discharge/charge curve. Reproduced with permission [44]. Copyright 2022, Wiley-VCH Verlag GmbH & Co. KGaA.

Simultaneously, the in situ conversion chemistry driven by Li^+^ was proposed to synthesize Bi anode from bismuth selenide [44]. The Bi (BS-Bi) anodes were fabricated by treating the solvothermal-synthesized Bi_2_Se_3_ nanosheets precursor. The electrolyte was the 0.1 M Mg(BH_4_)_2_-1.5 M LiBH_4_/diglyme (MLBH) hybrid electrolyte. The two-stage reaction mechanism was proposed in Figure 10a. The upper discharge plateau can be observed at approximately 0.8 V vs. Mg^2+^/Mg, related to the activation of BS-Bi electrode induced by Li^+^, while the lower plateau corresponds to the alloying reaction of Bi with Mg^2+^ (Figure 10b). The scanning electron microscopy (SEM) and TEM results depicted that the Bi_2_Se_3_ was composed of the randomly stacked hexagonal nanosheets (Figure 10c,d). The HRTEM image of the BS-Bi at the discharge state of 0.75 V vs. Mg^2+^/Mg confirms the in situ conversion process from Bi_2_Se_3_ to monoclinic Bi (m-Bi) nanocrystals (Figure 10e). Compared with the control sample (bismuth nanosheets (Bi NS)), the BS-Bi electrodes displayed an excellent rate performance (335 mAh g^−1^ at 1 A g^−1^) and long cycling stability (252 mAh g^−1^ over 600 cycles) (Figure 10f,g), demonstrating that the in situ formation of the nanosized Bi structure contributes to the fast Mg^2+^ diffusion kinetics and highly efficient alloying/dealloying processes of Mg-Bi. More importantly, operando synchrotron XRD was performed to unveil the reaction mechanism of BS-Bi in the MLBH electrolyte (Figure 10h). The stage I in the initial discharge process is associated with the Li^+^-driven in situ conversion from Bi_2_Se_3_ to metallic Bi (Bi_2_Se_3_ → BiSe → Bi_4_Se_3_ → m-Bi), followed by the reversible alloying/dealloying reaction of Bi with Mg^2+^ (The conversion from Bi to Bi_2_Se_3_ was very difficult during the charge process). Noticeably, the intermediate phase MgBi was also identified during the reversible (de)magnesiation process of Bi. Moreover, a new bismuth phase of orthorhombic Bi (o-Bi) was captured at the end of charge. The phase transition from m-Bi to o-Bi may be ascribed to the efficient alloying/dealloying processes stemming from the nanoscale of BS-Bi crystals. The in situ conversion procedure in this work could provide new ideas for exploring other high-performance electrode materials for batteries.

Based on all the above discussions, Bi-based anodes are distinguished for their rapid Mg^2+^ transport kinetics and good compatibility with conventional electrolytes, but the huge volume variations during discharge/charge processes need to be adequately addressed to achieve long-cycle stability at various rates. There are two main views on the reaction mechanism of Bi anodes towards Mg storage, namely Bi ↔ Mg_3_Bi_2_ and Bi ↔ MgBi ↔ Mg_3_Bi_2_, respectively, which can be further verified in more detail in subsequent studies.

## 4. Bi-Based Alloy-Type Anodes

Alloys containing two or more metallic elements tend to show superior electrochemical properties compared with monometallic hosts. The electrodeposited Bi_0.88_Sb_0.12_ and Bi_0.55_Sb_0.45_ films were prepared by Arthur et al. [23] and expected to improve the energy density of the anode via combining the low reaction potential of Bi with high theoretical capacity of Sb. However, the actual Mg storage performance of Bi-Sb films was inferior to that of the pure Bi anodes, which can be attributed to the high ionicity and strong Mg-Sb bond in Mg_3_Sb_2_. Furthermore, according to the discharge curves of Bi_1−x_Sb_x_ films and the reaction plateaus of Sb and Bi, the authors proposed that the magnesiation reactions of Sb and Bi occur at 0.27–0.29 and 0.23 V vs. Mg^2+^/Mg to form Mg_3_Sb_2_ and Mg_3_Bi_2_, respectively. Afterwards, Murgia et al. [45] have also investigated the Mg storage properties of the micrometric Sb_1−x_Bi_x_ solid solutions, which were synthesized by the high-energy ball milling method. The operando XRD and ^25^Mg solid-state NMR results depicted the one-step alloying process from Sb_1−x_Bi_x_ to monophasic Mg_3_(Sb_1−x_Bi_x_)_2_ upon discharge, differing from the two-step magnesiation mechanism of the electrodeposited Bi-rich alloys. Such synergistic reaction mechanism may facilitate electrochemical magnesiation process of electrodes and enable higher specific capacities. Nevertheless, this synergy was only limited to the first magnesiation process, while the irreversible capacity loss was observed in the subsequent demagnesiation process, which can be assigned to the formation of Bi and Mg_3_Sb_2_ at the end of charge. The Mg_3_Sb_2_ is highly stable, resulting in the difficulties for extracting Mg from Mg_3_Sb_2_. They have also synthesized the intermetallic InBi anodes for MIBs through high-energy ball-milling upon metallic In and Bi powders [46]. The discharged products of intermetallic InBi anodes are Mg_3_Bi_2_ and MgIn after magnesiation, and the InBi was reformed after complete demagnesiation. The operando XRD results revealed the complicated reaction procedure instead of the independent magnesiation reaction of Bi and In, involving various reversible immediate products of In_2_Bi and several Mg-In phases. However, the electrochemical performance of the InBi anodes was intermediate between those of pure Bi and In electrodes, failing to achieve the synergistic improvement between these two active elements. Additionally, the authors suggested that the formation of metallic In during the discharge/charge process may strongly influence the electrochemical performance of InBi electrode.

**Figure 11:**
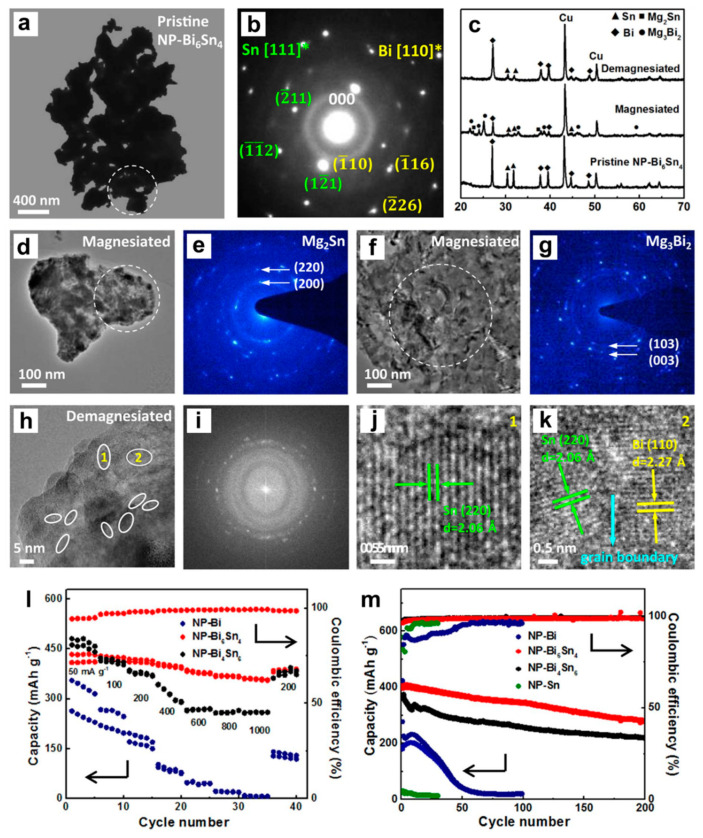
(**a**) TEM image and (**b**) the corresponding SAED pattern of pristine nanoporous Bi_6_Sn_4_ alloy. (**c**) Ex situ XRD patterns of the Bi_6_Sn_4_ electrode before and after the first discharge/charge. (**d**,**f**) TEM images and (**e**,**g**) the corresponding SAED patterns of the first magnesiated Bi_6_Sn_4_ electrode. The areas for electron diffraction are marked by white circles in (**a**,**d**,**f**). (**h**) HRTEM image of the first demagnesiated Bi_6_Sn_4_ electrode, (**i**) the corresponding FFT pattern and (**j**,**k**) the enlarged HRTEM images corresponding to regions 1 and 2 in (**h**). (**l**) Rate performance and (**m**) cycling stability of the nanoporous Bi, Bi-Sn and Sn electrodes. Reproduced with permission [47]. Copyright 2018, Elsevier B.V.

Subsequently, Niu et al. [47] developed the high-performance Bi-Sn alloy anodes (Bi_6_Sn_4_ and Bi_4_Sn_6_) for MIBs, which were prepared via chemical dealloying of rapidly solidified Mg_90_Bi_6_Sn_4_ (at%) and Mg_90_Bi_4_Sn_6_ (at%) precursor ribbons in a 2 wt% tartaric acid solution at ambient temperature. TEM results of Bi-Sn alloys show a typical nanoporous structure with high density phase boundaries (Figure 11a,b), which also confirmed the coexistence of Bi and Sn phases with interdigitated phase distribution feature. The Bi-Sn alloy anodes were tested in the half cells versus Mg metal as the counter/reference electrodes with the 0.4 M APC electrolyte. Additionally, the Mg storage mechanism of dual-Bi-Sn anodes was explored by the ex situ XRD and TEM characterizations (Figure 11c–k). The magnesiation process of dual-phase Bi-Sn involves the successive reaction of Bi and Sn with Mg^2+^ to generate Mg_3_Bi_2_ and Mg_2_Sn, respectively, and the charge process is related to the extraction of Mg^2+^ from Mg_2_Sn/Mg_3_Bi_2_ to reform smaller-sized Sn/Bi nanocrystals. More importantly, the dual-phase Bi-Sn anodes exhibit much enhanced Mg storage performance compared to their single-phase counterparts (Bi and Sn). Especially, the Bi_6_Sn_4_ electrode could deliver excellent rate capability (362 mAh g^−1^ at 1000 mA g^−1^) and good cycling stability (280 mAh g^−1^ after 200 cycles), as shown in Figure 11l,m, which could be attributed to the unique porous structure and increased phase boundaries, thus promoting Mg^2+^ transports and alleviating large volume variations. This work made a good start for the exploration and development of dual-phase electrodes in MIBs. In a follow-up work, they further demonstrated a scalable strategy to synthesize porous Bi-Sn alloys via the selective phase corrosion, where the compositions/sizes can be controlled through optimizing the triphase precursor (Al-Bi-Sn) composition based on the positive enthalpy of elemental mixing [48]. The experimental results revealed that the alloy composition and ligament size determined the Mg storage properties of the porous Bi-Sn anodes. Among all the electrodes with different compositions, the Bi_3_Sn_2_ anode delivered an outstanding reversible capacity retention of over 93% for 200 cycles at 1000 mA g^−1^. Further, operando XRD measurements further confirmed the reversible two-step processes of Mg storage for Bi-Sn anodes: 2Bi + 3Mg^2+^ + 6e^−^ ↔ Mg_3_Bi_2_, Sn + 2Mg^2+^ + 4e^−^ ↔ Mg_2_Sn.

**Figure 12:**
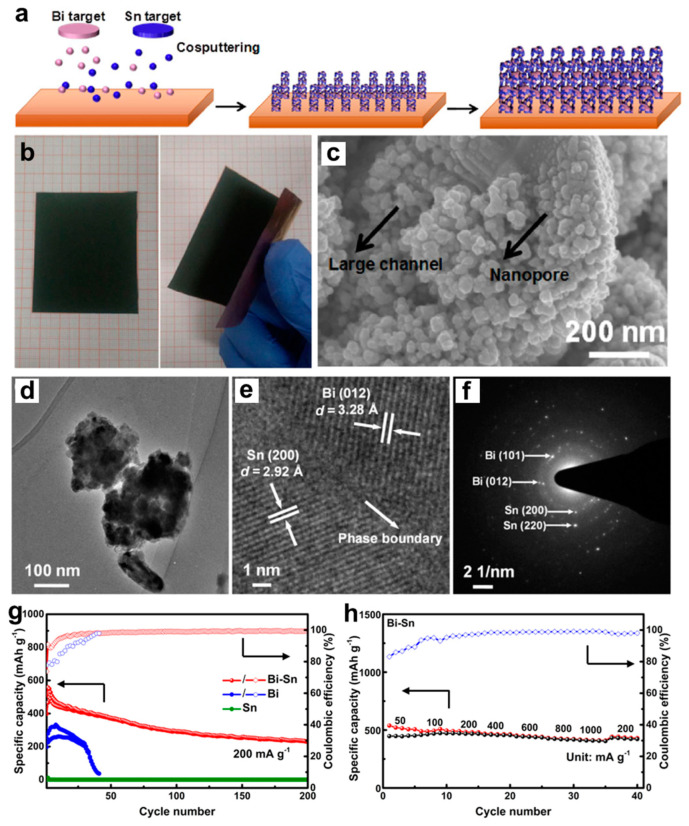
(**a**) Schematic illustrations showing the preparation procedure of the biphase eutectic-like Bi-Sn film. (**b**) Photographs of the sputtered Bi-Sn film showing its flexible, self-supporting, robust feature. (**c**) SEM, (**d**) TEM, (**e**) HRTEM and (**f**) corresponding SAED images of the biphase Bi-Sn film. (**g**) The cycling stability of the sputtered Bi, Bi-Sn electrodes at 200 mA g^−1^ and Sn electrode at 10 mA g^−1^. (**h**) Rate performance of the biphase Bi-Sn electrode at different current densities. Reproduced with permission [49]. Copyright 2019, Springer Nature.

On account of the favorable biphase structure in Bi-Sn alloys, Song et al. [49] have designed the biphase, eutectic-like Bi-Sn film anodes with interdigitated phase distribution and a hierarchically porous structure, which were fabricated through the facile one-step magnetron co-sputtering methods utilizing the Bi and Sn targets and Cu foil substrate (Figure 12a). The flexible, self-supporting Bi-Sn films without any binder and conductive agent could be directly used as the working electrodes (Figure 12b). SEM and TEM images suggest the formation of a hierarchically porous structure containing large channel and nanopore in the sputtered Bi-Sn film (Figure 12c,d). As indicated in the HRTEM image (Figure 12e), obvious lattice fringes belonging to Bi and Sn could be observed, and there are distinct phase boundaries between Bi and Sn two phases. The corresponding SAED pattern (Figure 12f) shows spotty rings of Bi and Sn, confirming the co-existence of Bi and Sn phases. The thickness of Bi-Sn films is approximately 1.3 μm. These biphase Bi-Sn anodes could deliver high specific capacity (538 mAh g^−1^ at 50 mA g^−1^), good cycling stability (233 mAh g^−1^ at 200 mA g^−1^ after 200 cycles) and excellent rate performance (417 mAh g^−1^ at 1000 mA g^−1^), compared to the single-phase Bi or Sn film (Figure 12g,h). According to mass loading (0.85 mg cm^−2^) and electrode area (1.13 cm^2^) of Bi-Sn films, the areal specific capacity of Bi-Sn electrode at 1000 mA g^−1^ can be converted to approximately 0.35 mAh cm^−2^ (Figure 12h), which is a relatively high areal specific capacity value for batteries. Such remarkable performance benefits from the synergetic effect of the hierarchically porous structure, interdigitated Bi/Sn phase distribution and increased phase boundaries. More remarkably, the specific capacities of Bi-Sn film electrodes can be maintained at 239 and 182 mAh g^−1^ at 1000 and 3000 mA g^−1^ after 200 cycles, respectively. This work can provide an important reference for the large-scale fabrication of biphase electrodes in batteries. Afterwards, they further introduced the second phase of Bi with varying contents into Sn through magnetron co-sputtering and explored how the introduction of Bi triggers the electrochemical reactivity of Sn by combining experiments and DFT calculations [50]. The experimental results of the sputtered Sn-Bi films demonstrated that the introduction of Bi can effectively boost the electrochemical reaction of Sn with Mg, and the further increasing of Bi content can significantly enhance the Mg storage performance (cycling stability and rate performance) of Sn-Bi electrodes. Based on the DFT calculations, the existence of second phase Bi or Mg_3_Bi_2_ (first formed during the magnesiation process of Sn-Bi electrode), as well as the tiny amount of Bi solid solution in Sn, can significantly lower the defect formation energy of Mg insertion into Sn phase. Hence, the introduction of Bi can effectively stimulate the electrochemical reactivity of Sn towards Mg storage. Further, the bulk rolled (micro-sized) Sn-Bi system further revealed the activated function of introducing Bi into Sn, implying that the size effect might not be the decisive factor affecting the electrochemical performance of Sn. These studies suggest that the active element Bi may have a positive effect on unlocking the potential of Mg storage for other less active elements.

**Figure 13:**
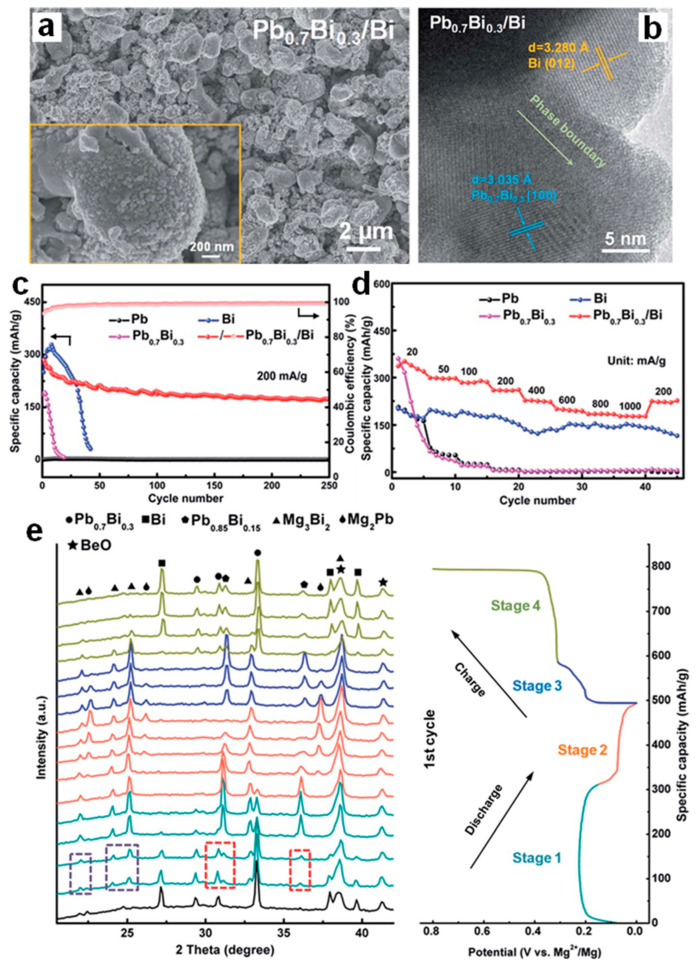
(**a**) SEM and (**b**) HRTEM images of the biphase Pb_0.7_Bi_0.3_/Bi film. (**c**) Cycling stability and (**d**) rate performance of the sputtered Pb, Bi, Pb_0.7_Bi_0.3_ and Pb_0.7_Bi_0.3_/Bi electrodes. (**e**) Operando XRD results of the sputtered Pb_0.7_Bi_0.3_/Bi film during the first discharge/charge processes at 20 mA g^−1^. Reproduced with permission [51]. Copyright 2020, The Royal Society of Chemistry.

**Figure 14:**
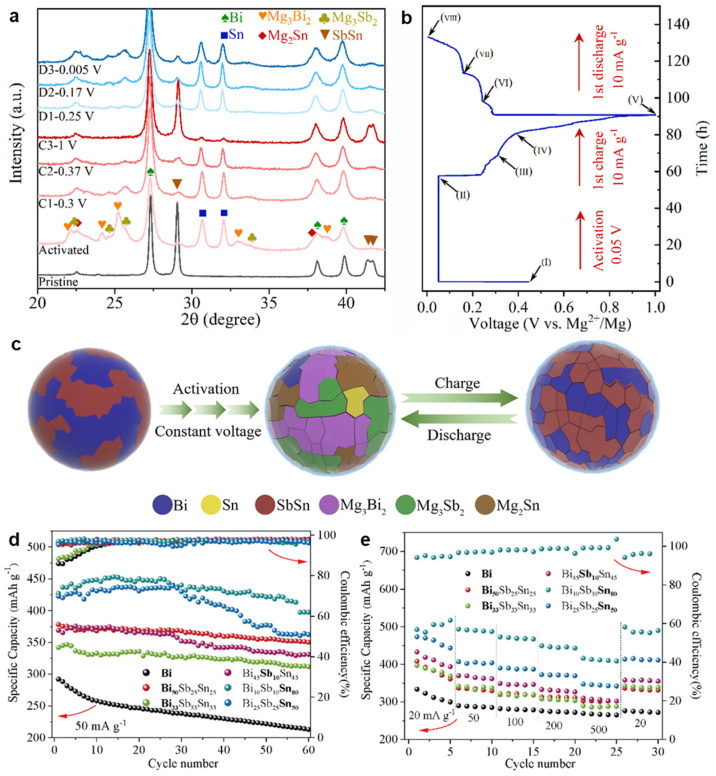
(**a**) The ex situ XRD patterns of the Bi_33_Sb_33_Sn_33_ electrode at the varied states in cycling. (**b**) The discharge/charge curve of Bi_33_Sb_33_Sn_33_ electrode at 10 mA g^−1^ corresponding to (**a**). (**c**) Schematic illustration of the electrochemical reaction mechanism of Bi_33_Sb_33_Sn_33_ particles during the discharge/charge process. (**d**) Cycling stability and (**e**) rate performance of the Bi and Bi-Sb-Sn anodes. Reproduced with permission [52]. Copyright 2022, Elsevier B.V.

Inspired by the significantly improved electrochemical performance of biphase Bi-Sn electrodes and based upon the phase engineering strategy, Song et al. [51] have further designed and fabricated the biphase Pb_0.7_Bi_0.3_/Bi films as MIB anodes through the facile magnetron co-sputtering route. As shown in the SEM image (Figure 13a), the micro-sized pillars composed of nanoparticles with the diameter of less than 100 nm, formed the biphase Pb_0.7_Bi_0.3_/Bi film with the porous structure. The HRTEM result (Figure 13b) confirms the co-existence of two Pb_0.7_Bi_0.3_ and Bi phases with noticeable phase boundaries between them. As benchmarked with single-phase Pb, Bi and Pb_0.7_Bi_0.3_ films, the biphase Pb_0.7_Bi_0.3_/Bi electrode exhibits greatly enhanced cycling stability (171.8 mAh g^−1^ at 200 mA g^−1^ after 250 cycles) and rate capability (177.4 mAh g^−1^ at 1000 mA g^−1^, as presented in Figure 13c,d. Operando XRD experiments combined with DFT calculations revealed the Mg storage mechanisms of the Pb_0.7_Bi_0.3_ and Pb_0.7_Bi_0.3_/Bi electrodes. Both of them follow a two-step alloying process with the final discharge products of Mg_3_Bi_2_ and Mg_2_Pb, and an intermediate phase Pb_0.85_Bi_0.15_ was identified during the magnesiation/demagnesiation processes (Figure 13e). Further, the biphase Pb_0.7_Bi_0.3_/Bi anodes show good compatibility with the Mg(TFSI)_2_ conventional electrolytes, further confirming the practical application potential of Pb-Bi alloy anodes in MIBs. Recently, Gu et al. [52] have studied the electrochemical properties of Bi-Sb-Sn alloy anodes with varied compositions for MIBs, which were synthesized via the mechanical alloying method. The ex situ XRD results of the Bi_33_Sb_33_Sn_33_ electrodes demonstrated that the Sn substitution for Mg in Mg_3_Sb_2_ to form SbSn during the charge process improved reversible Mg storage of Sb (Figure 14a,b). The detailed Mg storage mechanism of Bi_33_Sb_33_Sn_33_ is illustrated in Figure 14c. After activation, the Bi_33_Sb_33_Sn_33_ alloy particles cracked and transformed into smaller particles, facilitating the reaction kinetics and achieving subsequent stable cycling. Benefiting from the special multi-phase structures, interfaces and substructures, the Bi-Sb-Sn anodes showed much better cycling stability and rate performance than Bi anodes (Figure 14d,e). In particular, the Bi_10_Sb_10_Sn_80_ anode could deliver a high discharge capacity of 517 mAh g^−1^ at 20 mA g^−1^ and an excellent rate capacity of 417 mAh g^−1^ at 500 mA g^−1^. This work further confirmed the importance of phase design for improving the electrochemical performance of electrodes.

## 5. Summary and Outlook

Using alternative alloy-type anode materials is an efficient strategy to circumvent the passivation issue of Mg metal anodes in conventional electrolytes and construct the high-performance MIB system by pairing with the high voltage cathodes. Among them, Bi-based anodes have shown great application potential due to their high electrochemical reactivity and fast transport kinetics of Mg^2+^, which were confirmed by some theoretical simulations. In order to alleviate the capacity attenuation caused by significant volume variations of Bi anodes, many effective strategies were adopted, including structural modifications (nanotubes, nanowires, nanocrystals, thin films, etc.) and composite materials (Bi/RGO, Bi-CNT, BiOF, Bi@NC, etc.) Additionally, the greatly enhanced electrochemical performance towards Mg storage of these Bi-based anodes could be obtained. Further investigations on Mg_3_Bi_2_ alloy anodes and their excellent Mg storage performance also substantiated the importance of Bi-based materials as anodes for MIBs. Further, bi/muti-metallic Bi-based alloy anodes (Bi-Sb, In-Bi, Bi-Sn, Pb-Bi, Bi-Sb-Sn, etc.) can serve as the promising hosts for Mg storage and showed superior properties. Simultaneously, Bi serving as a fast Mg^2+^ transport medium can promote the electrochemical reactivity of other less active elements. Moreover, Bi-based materials exhibited well compatibility with conventional electrolytes. For Mg storage mechanism of Bi anodes, most studies demonstrated the simple two-phase reaction between Bi and Mg_3_Bi_2_. Interestingly, the reversible two-step (de)alloying reaction of Bi ↔ MgBi ↔ Mg_3_Bi_2_ has recently been proposed and the intermediate phase MgBi could promote the Mg^2+^ diffusion and mitigate volume expansion of Bi anodes, which provides an important reference for further understanding and exploring the reaction mechanism of Bi.

Actually, more comprehensive and deep investigations are needed to further improve the Mg storage properties of Bi-based anodes, such as the interface modification between active Bi materials and current collectors, in/ex situ exploration for the Bi-based electrode/electrolyte interface reaction mechanism, and investigation of more optimized Bi-based multi/bi-metallic hosts. By rationally designing the structure of electrodes and optimizing the electrode/electrolyte system, the achievement of a high-capacity, long-lifespan, and high-safety MIB system will be a major step forward.

## Data Availability

Not applicable.
